# Helically structured metal–organic frameworks fabricated by using supramolecular assemblies as templates†
†Electronic supplementary information (ESI) available: Detailed TEM images and other extensive figures. See DOI: 10.1039/c4sc03278k



**DOI:** 10.1039/c4sc03278k

**Published:** 2014-12-23

**Authors:** Hui Wang, Wei Zhu, Jian Li, Tian Tian, Yue Lan, Ning Gao, Chen Wang, Meng Zhang, Charl F. J. Faul, Guangtao Li

**Affiliations:** a Department of Chemistry and Key Lab of Organic Optoelectronics & Molecular Engineering , Tsinghua University , Beijing , 100084 , P. R. China . Email: lgt@mail.tsinghua.edu.cn ; Tel: +86-010-62792905; b School of Chemistry , University of Bristol , Cantock's Close , Bristol BS8 1TS , UK

## Abstract

The controlled formation of MOF-based superstructures with well-defined nanoscale sizes and exquisite morphologies represents a big challenge, but can trigger a new set of properties distinct from their bulk counterparts. Here we report on the use of a self-assembled organic object to template the first example of a nanoscale metal–organic framework (MOF) with a helical morphology. Two prototypical MOFs (**HKUST-1** and **MIL-100**) were used to exemplify the growth of such materials on supramolecular assemblies. Interestingly, it was found that, dependent on the nature of the precursors, not only could well-defined helical MOF nanotubes be facilely fabricated, but novel helical bundle nanostructures could also be formed. These resultant MOF superstructures show additional optical properties and could be used as precursors for the preparation of chiral nanocarbons.

## Abstract 2

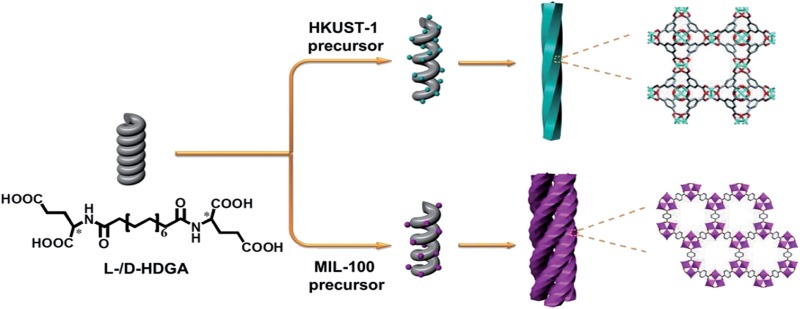
Here we report on the use of a self-assembled organic object to template the first example of a nanoscale metal–organic framework (MOF) with a helical morphology.

## Introduction

Metal–organic frameworks, also known as coordination polymers, are a fascinating class of hybrid porous crystalline materials made by linking metal-containing units with organic linkers using strong bonds.^1^ Their unique features, including high porosity, large internal surface area, intricate porous structure, diversity and tailorable chemistry, make them very attractive for numerous applications.^2^ Nanoscale metal–organic frameworks (NMOFs), where the sizes of well-defined MOFs are reduced to micro- and nano-scales, show improved properties when compared to their bulk counterparts.^3^ Their potential applications can be significantly extended owing to the superior adsorption kinetics of guest species and their dispersible nature which is distinct from bulk crystals.^3^ Keeping these attractive features in mind, increasing efforts have been devoted to reducing the size of MOF crystals to the nanometer scale, while retaining their well-defined morphologies. A few approaches, including micro-emulsion, microwave synthesis and methods using capping agents *etc.*, have been developed for the fabrication of nanometer-sized MOFs,^3^ but the nanoscale morphologies accessible to date have been largely limited to fairly basic shapes, such as nanoparticles, hollow nanospheres, nanocubes, nanorods, nanosheets, and core–shell nanostructures.^3,4^ However, more complex morphologies like helices, which have promising potential applications in, for example, asymmetric catalysis, helical sensors and optical devices,^5^ have not been described for MOF materials. Conceivably, controlled fabrication of hierarchical MOF-based superstructures with helical morphologies and permanent porosity would create new opportunities for the development of advanced applications owing to the combination of the intrinsic MOF properties and the nanostructure-related optical activity.

Amphiphilic molecules have long been known to self-assemble into a variety of ordered supramolecular nanostructures.^6^ An intriguing aspect of amphiphiles is that the sizes, morphologies and surface chemistry of the resulting organized supramolecular assemblies are easily tunable by modulation of the molecular structure and the experimental conditions. Beyond the common micelles, vesicles and liquid crystalline mesophases, more stable and organized nanostructures such as fibers, ribbons, helices, super helices and tubes can be facilely achieved. The ability of amphiphilic molecules to form such self-assembled objects with well-defined sizes and designed morphologies implicates the great potential of supramolecular assemblies as templates for the controlled production of nanostructured materials that are inaccessible by conventional methods. In this respect, the templating power of supramolecular assemblies was exemplified by the transcription synthesis of metals, metal oxides, metal chalcogenides and silicates.^7^ For example, Hanabusa and Shinkai reported the creation of helically nanostructured silicas by using sol–gel reaction in the presence of diaminocyclohexane-based chiral organogels.^8^ Stupp *et al.* have also successfully fabricated single and double helices of cadmium sulfide (CdS) fibers from supramolecular assemblies.^9^ However, most of the reported works demonstrated the creation of helically nanostructured silicates, cadmium sulfide, metals or metal oxides by using supramolecular assemblies as templates.^7–9^ To the best of our knowledge, there have been no reports on the direct growth of MOF crystals on the surface of discrete supramolecular assemblies to form helical MOF nanomaterials.

**Scheme 1:**
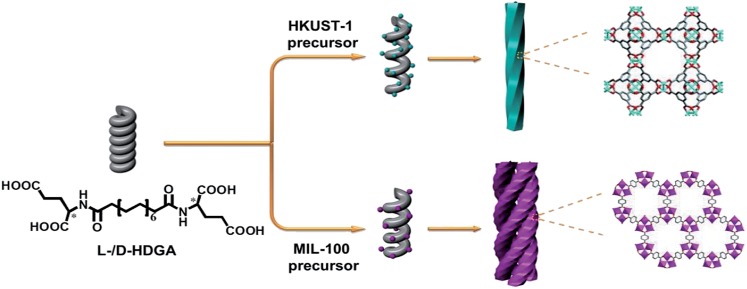
Schematic illustration of the fabrication of helical MOF nanostructures by using supramolecular assemblies as templates.

In this study, we therefore present the first example of the preparation of helically structured MOF nanomaterials from supramolecular assemblies of two low-molecular-weight bolaamphiphilic templates in a controlled manner. As shown in Scheme 1, two bolaamphiphiles based on either d- or l-glutamic acid were synthesized and used to form well-defined helical supramolecular aggregates. As the surfaces of the aggregates consist of hydrophilic carboxyl groups, it is expected that the helical surfaces should show strong affinities for metal cations, and thus enable the nucleation, crystallization and growth of MOFs on the curved surfaces of the helical aggregates. To prove our concept, a prototypical MOF (**HKUST-1**) was used as example. Indeed, we found that after optimization of the preparation conditions, the chirality of the helical supramolecular aggregate could be reliably expressed onto the formed **HKUST-1**, affording well-defined, helically nanostructured **HKUST-1**. d- and l-type helical NMOF products could be selectively created, depending on the chirality of the supramolecular template used. Moreover, the pitch of the resultant helical MOF could also be well adjusted from 170 nm to 305 nm by controlling the growth time of **HKUST-1**. To further examine the validity and general applicability of our controlled fabrication strategy for NMOFs with helical morphologies, another well-studied MOF (**MIL-100**) was also chosen for the production of helical NMOF constructs under similar conditions. As expected, similar results as for **HKUST-1** were obtained, and nanoscale **MIL-100** with a helical morphology was also produced. However, probably due to the stronger affinity of the carboxyl groups for Fe^3+^ (compared with Cu^2+^), more complex helical bundle superstructures were achieved through intertwining of a few helical **MIL-100** nanofibers. These resultant NMOF (**HKUST-1** or **MIL-100**) superstructures showed additional optical properties and could be used as precursors for the preparation of chiral nanocarbons.

## Experimental

### Preparation of helical self-assembled templates

To prepare helical self-assembled templates, 2.7 mg **l-HDGA** solid was added into 0.5 mL pure water, sealed and heated to 100 °C until fully dissolved. The solution was then cooled to room temperature and a viscous gel was formed after 10 minutes. TEM samples were prepared by suspending a portion of the **l-HDGA** hydrogel in H_2_O, casting it onto a holey carbon coated Cu grid, and blotting off the redundant liquid with filter paper. The **d-HDGA** hydrogel could be fabricated and prepared for imaging in the same way.

### Preparation of helical **HKUST-1** (**l-/d-HDGA@HKUST-1**)

Typically, to prepare helical **l-HDGA@HKUST-1**, 0.5 mL **l-HDGA** hydrogel was diluted with 4 mL of mixed solvent (0.4 mL ethanol and 3.6 mL pure water) and stirred for 1 hour to ensure homogeneous dispersion of the self-assembled templates in the mixed solution. Then, 400 μL of fresh MOF precursor solution and 200 μL of 50 mM Cu(NO)_3_ ethanol solution, which was mixed with 200 μL of 25 mM H_3_BTC (1,3,5-benzenetricarboxylic acid) ethanol solution, were fed into the prepared **l-HDGA** solution under magnetic stirring. After incubation under static conditions at 26 °C for 24 h, the as-formed helical **l-HDGA@HKUST-1** was collected by centrifugation. **d-HDGA@HKUST-1** could be fabricated based on the same methodology.

***n*-l-HDGA@HKUST-1** (*n* = 1, 2, 3, 4) with different shell thicknesses (from thinner to thicker: **1-l-HDGA@HKUST-1**, **2-l-HDGA@HKUST-1**, **3-l-HDGA@HKUST-1** and **4-l-HDGA@HKUST-1**) could be obtained by adding MOF precursor solutions dropwise. **1-l-HDGA@HKUST-1** could be fabricated by feeding 200 μL of fresh MOF precursor solution (100 μL of 50 mM Cu(NO)_3_ ethanol solution mixed with 100 μL of 25 mM H_3_BTC ethanol solution) into the diluted **l-HDGA** hydrogel solution under magnetic stirring. The mixed solution was incubated under static conditions at 26 °C for 24 h. **2-l-HDGA@HKUST-1**, **3-l-HDGA@HKUST-1** and **4-l-HDGA@HKUST-1** could be obtained by repeating the procedure for **1-l-HDGA@HKUST-1** two, three or four times, respectively. ***n*-d-HDGA@HKUST-1** (*n* = 1, 2, 3) could be fabricated based on the same methodology, but using **d-HDGA** hydrogel as the template.

### Preparation of helical **MIL-100** (**l/d-HDGA@MIL-100**)

Typically, to prepare helical **l-HDGA@MIL-100**, 200 μL of fresh MOF precursor solution (100 μL of 40 mM FeCl_3_ ethanol solution mixed with 100 μL of 40 mM H_3_BTC ethanol solution) was fed into a diluted **l-HDGA** hydrogel solution under magnetic stirring, and the mixed solution was incubated under static conditions at 26 °C for 24 h. Subsequently, the above procedure was repeated once more. The formed helical **l-HDGA@MIL-100** could then be collected by centrifugation. **d-HDGA@MIL-100** could be fabricated in the same way as **l-HDGA@MIL-100**, except that **d-HDGA** hydrogel was used as the template.

### Preparation of helical carbon nanotubes

To prepare helical carbon nanotubes, the helical ***n*-l-HDGA@HKUST-1** (*n* = 1, 2, 3) was calcined under N_2_ atmosphere. The annealing procedure was performed at a heating rate of 5 °C min^–1^ and kept at 600 °C for 0.5 hour, and then allowed to cool naturally to room temperature. After that, helical carbon nanotubes could be obtained.

## Results and discussion

**Fig. 1:**
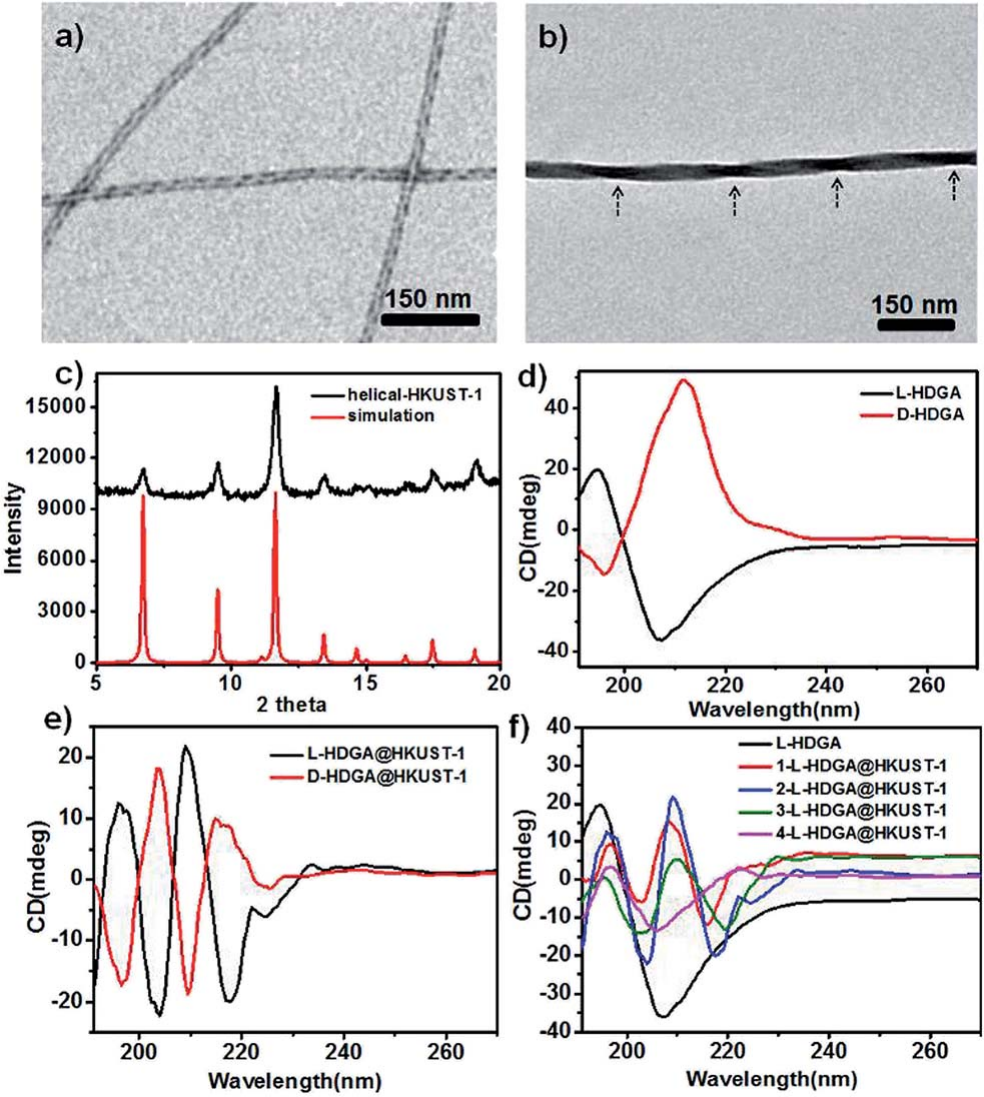
TEM images of right-handed helices prepared from self-assembly of **l-HDGA** (a) and the as-prepared right-handed helical **HKUST-1** (b); XRD patterns of the resultant helical **HKUST-1** and bulk **HKUST-1** nanocrystals (c); CD spectra of the **l-HDGA** and **d-HDGA** based templates (d); CD spectra of the resulting right/left-handed helical **HKUST-1** (e); evolution of the CD spectrum of the right-handed helical **HKUST-1** prepared by increased addition of the MOF precursor (f).

l- or d-glutamic acid based bolaamphiphiles (**l-HDGA** or **d-HDGA**) used here were synthesized according to a reported procedure.^10^ These compounds formed helical nanotubes during self-assembly in water. Fig. 1a and S1† show TEM images of the formed supramolecular aggregates at different magnifications, which clearly indicate a single helical nanotube structure. With a pitch of about 40 nm, diameter of 16 nm and length of several micrometers, the handedness of the nanotubes is determined by the chirality of the bolaamphiphile. While **l-HDGA** forms right-handed helical tubes, **d-HDGA** forms left-handed tubes. Initially, to examine the possibility of producing helical MOF nanostructures by using HDGA supramolecular assemblies as templates, we added a **HKUST-1** precursor solution directly to the **l-HDGA** aggregates (hydrogel) system. After a fixed period (12 h) of crystallization and growth of **HKUST-1**, we found that the organized carboxyl groups on the curved surfaces of the formed assemblies could function as anchors to absorb metal ions for the nucleation, growth and deposition of **HKUST-1**, affording MOF nanotubes. However, the formed tubes contained a lot of cracks dispersed across their surface (Fig. S2†), most probably because of the overlap of the HDGA assembled aggregates due to the viscous nature of the hydrogel, which hindered complete coating of the MOF on the surface of the supramolecular template. We therefore decided to use a diluted suspension of HDGA aggregates in a water–ethanol mixture (instead of in the gelled state), to correctly implement the transcription of the HDGA-based template.

Hydrogels containing 0.5 wt% HDGA were first prepared and then diluted with an ethanol–water mixture to form suspensions of HDGA aggregates (see below). The addition of ethanol is favorable for the dissolution of organic ligands, which is in turn necessary for the formation of MOF crystals. However, excess ethanol would diminish the inter- or intramolecular interactions (especially the hydrogen bonding) in the highly organized supramolecular aggregates, leading to change in the formed morphology, or even destruction of the well-defined supramolecular aggregates.^11^ The evolution of the supramolecular assemblies was then systematically investigated with stepwise increases in the ethanol content from 0 to 40 vol%. As shown in Fig. S3,† both the diameters of the HDGA nanotubes and the pitches of the helical tubes become larger with the gradual increase in ethanol content in the mixture. Based on these results, a water–ethanol mixture containing 10 vol% ethanol was used to dilute the HDGA hydrogel. The MOF nanomaterial (**l-HDGA@HKUST-1**) was successfully fabricated under these conditions, showing well-defined sizes and the exquisite helical morphology. Fig. 1b shows a representative TEM image of the as-prepared **l-HDGA@HKUST-1**, displaying a distinctive right-handed helical structure, which is consistent with the chirality of the **l-HDGA** template used. The outer diameter of the as-prepared MOF helix was 40 nm and the pitch of the outer surface along the rod axis was estimated to be approximately 200 nm, as indicated by the arrows. The crystal structure of **HKUST-1** deposited on the **l-HDGA**-based aggregates was confirmed by powder X-ray diffraction (Fig. 1c).^12^ Consistent with the TEM observation, the chiral conformations of the **d-/l-HDGA** templates and the resulting helical **d-/l-HDGA@HKUST-1** nanomaterials were clearly detected using a circular dichroism (CD) spectrometer (Fig. 1d and e). In the case of the diluted **l-HDGA** hydrogel suspension, positive and negative Cotton effects were observed at 195 nm and 205 nm, respectively, with a crossover at 198 nm, which is close to the absorption band of **l-HDGA** (Fig. S4a†). However, for the **l-HDGA@HKUST-1** sample, a new exciton-coupling band was detected in the range 205–235 nm with a crossover at 213 nm, which is assigned to a ligand-to-metal charge transfer (LMCT) transition from BTC^3–^ to Cu^2+^.^13^ This result indicates that the deposited **HKUST-1**, which possesses an achiral framework, inherits the chirality of the helical template and so shows CD activity in the ultraviolet wavelength region.

It should be noted that the conventional MOF **HKUST-1** microcrystal exhibits two absorption bands: the ligand-to-metal charge transfer (LMCT) transition with an edge at around 280 nm and the d–d transition of the Cu^2+^ at *ca.* 750 nm (Fig. S4b†).^13,14^ Unfortunately, we found that the formed helical **HKUST-1** nanowires dispersed in a water–ethanol mixture only show the LMCT transition (Fig. S4a†). However, the absorption band associated with the d–d transition of Cu^2+^ in the visible light region is too weak to be detectable. The exact reason is still not clear. The possible mechanism behind this phenomenon is owing to coordination of some solvent molecules to the metal (Cu^2+^) in the axial direction, which induces symmetry enhancement around the copper centre. As a result, the molar absorptivity (*ε*) associated with the d–d transition of Cu^2+^ is significantly reduced.^13,14^ In fact, we also found that a suspension of the conventional **HKUST-1** nanocrystals in a water–ethanol mixture didn't exhibit the absorption band associated with the d–d transition of Cu^2+^ in the visible light region (Fig. S4a†). Thus, the CD signal corresponding to this absorbance band (d–d transition of Cu^2+^) in the visible light region is absent for the suspension of the formed helical MOF wires. Similar results were also observed for the case of **d-HDGA@HKUST-1**. As shown in Fig. 1d and e, the mirror-imaged CD signals clearly confirm the antipodal left- and right-handed helical templates (Fig. 1d) and the resulting helical MOFs (Fig. 1e) with enantiomeric morphologies.

In our work, the helical MOF wires were prepared in a diluted suspension of the formed supramolecular assemblies. An increase in the concentration of the supramolecular assemblies or MOF precursor could lead to ill-defined MOF wire products or massive MOF nanocrystals due to the competitive growth of MOF crystals in the bulk solution. At present, it is still difficult to produce a large enough amount of the helical MOF wires for corresponding measurements on the solid samples. We are now focused on the development of new methods or approaches to solve the aforementioned problem. We hope that in the near future we will be able to perform UV-vis and CD spectral measurements on the helical MOF wire solid samples, which are expected to produce signals in the visible light region.

**Fig. 2:**
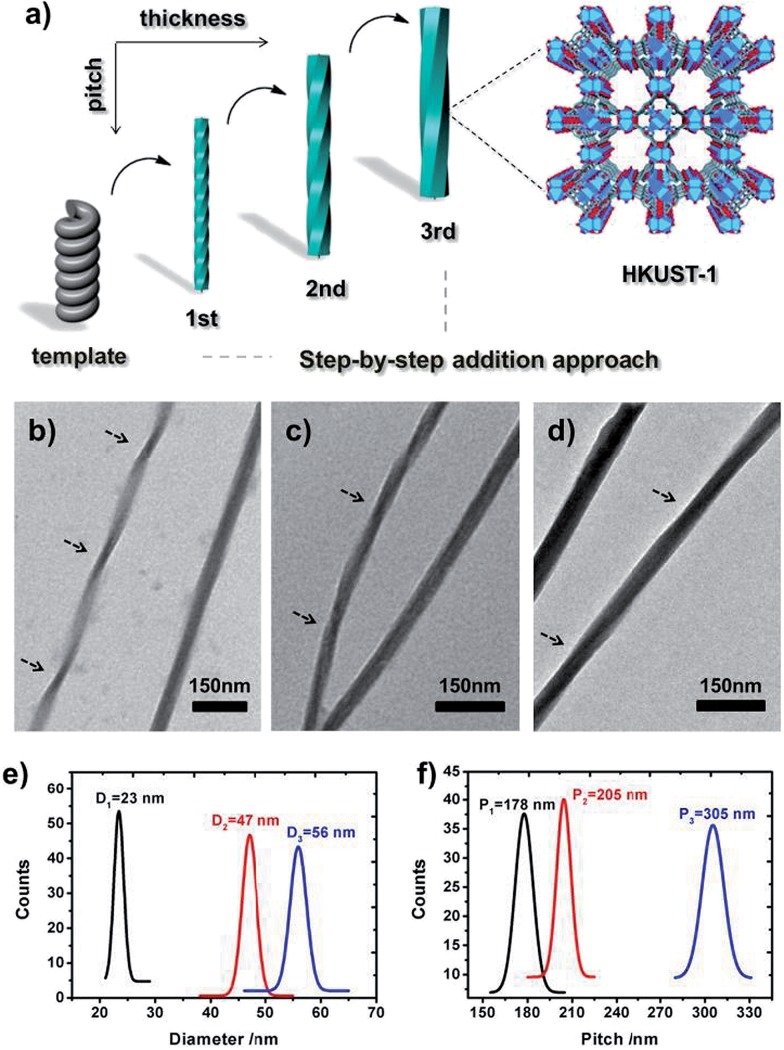
Schematic illustration of the fabrication of helical MOFs (**HKUST-1**) with different thicknesses and pitches based on a step-by-step addition approach (a); TEM images (b–d) of the resultant helical ***n*-l-HDGA@HKUST-1** (*n* = 1, 2, 3) nanomaterials with increased addition of the MOF precursor; evolution of the diameter (e) and pitch (f) of the resultant helical ***n*-l-HDGA@HKUST-1** (*n* = 1, 2, 3) nanomaterials with increased addition of the MOF precursor.

To fine-tune the morphology of the final products, step-by-step addition of fresh **HKUST-1** precursor solution into a suspension of HDGA aggregates was performed. As shown in Fig. 2, with stepwise addition of the **HKUST-1** precursor, the diameters of the resultant **l-HDGA@HKUST-1** nanomaterials gradually increased from 23 nm to 56 nm, and the pitches of the corresponding products varied from 178 nm to 305 nm. In our case, all of the diameters and pitches were manually measured from TEM images. These results indicate that by controlling the amount of MOF precursor added into the template suspension, the thickness and pitch of the resultant MOF nanomaterial can be finely adjusted. Helical MOF nanotubes with thick walls could be facilely fabricated using this approach, which might prevent the collapse of NMOFs during the removal of organic templates and provide opportunities for further practical use. Similar to the case of **l-HDGA**, by using **d-HDGA** aggregates as templates, helical **d-HDGA@HKUST-1** nanomaterials with exclusively left-handed chirality were also obtained (Fig. S8†). These structures, obtained from **d-HDGA** templates, are mirror images of the helical **l-HDGA@HKUST-1** nanostructures. A series of left-handed helical **HKUST-1** MOFs with different thicknesses and pitches were achieved through a similar procedure as that described above (Fig. S8†).

To reveal the evolution of the helical NMOFs, circular dichroism spectroscopy was used to detect the LMCT transition during the formation process. As shown in Fig. 1f, with the stepwise addition of the **HKUST-1** precursor into the **l-HDGA** template suspension, the CD signal of the **l-HDGA** template decreased gradually, while the CD signal assigned to the LMCT transition from BTC^3–^ to Cu^2+^ was enhanced for the first two addition procedures. This enhanced absorption of the LMCT process (Fig. S6†) could be attributed to the increased thickness of the MOF shells. With further addition of the **HKUST-1** precursor, the pitches of the resulting helical MOFs got longer and finally disappeared (Fig. 2d and S5†), resulting in the CD signals assigned to the LMCT transition being weakened and/or having disappeared. Fig. S6 and S7† show the evolution of the UV-vis spectra of the reaction system with different MOF precursor concentrations and MOF crystal deposition times, respectively. It was found that in both cases the absorption band of the as-prepared helical MOF nanowires only changed in intensity, while the location remained unchanged. This result indicates that the coordination of the carboxylate groups on the template surface with metal ions hardly exerts a marked influence on the structure of the deposited MOF material. The MOF deposited on the template surface has the same structure as that of the conventional MOF. Thus, variations in the LMCT and d–d transitions within the helical conformation were not detected in our case.

The preferred affinity of the metal ions (Cu^2+^) for the carboxyl groups of the organized HDGA assembled surface, which serves to initiate nucleation and subsequent growth of the MOFs along the curved surface of the assembly, is believed to be responsible for the formation of the observed helical NMOFs. However, as shown in Fig. 2b–d, the pitches of all of the products are clearly longer than those of their HDGA templates. To gain insight into the formation of the helical MOF nanotubes under the chosen reaction conditions, aqueous solutions containing different amounts of ethanol (0, 10, 20 and 30 vol%) were respectively added to the **l-HDGA** hydrogel. CD measurements were performed on the resultant suspensions to reveal the changes in the molecular packing of the self-assembled templates. It was found that with increasing concentrations of ethanol, the CD signal intensity of the **l-HDGA**-based supramolecular aggregates decreased gradually (Fig. S9†). This result implies that the presence of ethanol could influence the inter- or intramolecular interactions (especially the hydrogen bonding) of the HDGA assembled aggregates, loosening the molecular packing of the amphiphiles and stretching the pitch of the HDGA helical tubes in the water–ethanol solution. Thus, compared with the dried samples prepared for TEM observation, the supramolecular templates suspended in water–ethanol solutions should have longer pitches. Additionally, the nucleation and growth of MOF nanocrystals will probably fill up the spaces between the helices, leading to further broadening of the pitch of the formed helical NMOFs.

To demonstrate the “template” effect of the HDGA supramolecular aggregates, a high polarity solvent (methanol) was chosen to remove the HDGA assemblies from the as-prepared helical products. It should be noted that for products with thin MOF shells, removal of the template resulted in the collapse of the MOF superstructures. However, for the products with thicker MOF shells, the HDGA template could be removed in methanol within a few minutes, yielding nanotubes with a helical morphology (Fig. S10†). The inner diameter of the nanotubes is approximately 18 nm, which is consistent with the diameter of the HDGA helical aggregates used. This result clearly demonstrates the templating effect of the HDGA assemblies for the formation of helical NMOFs.

**Fig. 3:**
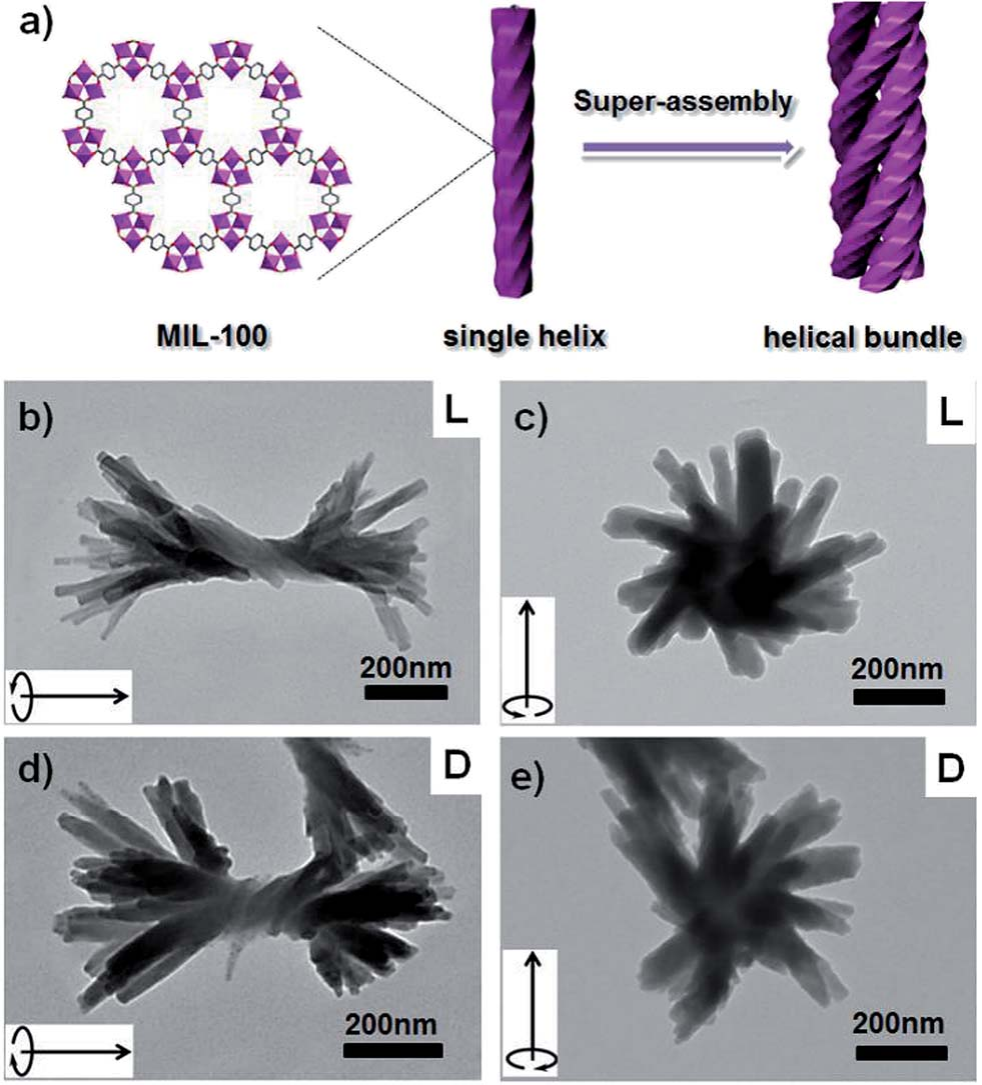
Schematic illustration of the formation of the hierarchical superstructure (a); TEM images of the resulting right-handed (b and c) and left-handed (d and e) hierarchical **MIL-100** superstructures. The arrow in the inset of each TEM image is to assist in the identification of the direction of the helicity of the formed superstructure.

To further demonstrate the validity and general applicability of the above mentioned transcription scheme, another prototypical MOF (**MIL-100**) was chosen for the production of **MIL-100**-based helices. A procedure similar to that used for the helical **HKUST-1** nanomaterials was adopted, with the exception that FeCl_3_·6H_2_O was used instead of Cu(NO_3_)_2_ in the preparation. Similar phenomena and results were observed. The seeding and subsequent growth of **MIL-100** on the surface of **l-HDGA** or **d-HDGA** based supramolecular aggregates afforded a large number of single helical **MIL-100** nanostructures (Fig. S11†). Interestingly, the well-defined helical **MIL-100** nano-objects could further self-assemble to form bundles of helical nanostructures, exhibiting a further higher level of organization (Fig. 3), most probably due to the high coordination valency and high affinity of Fe^3+^ relative to Cu^2+^. Furthermore, we found that the formed hierarchical superstructures inherited the chiralities of their respective HDGA templates at different length scales (helical wires and helical bundles), as shown in Fig. 3b–e. At present, the formation mechanism for these hierarchical superstructures is still not clear. Nevertheless, the obtained findings have already shown the power of the supramolecular aggregate transcription strategy for efficiently accessing NMOFs with novel morphologies.

**Fig. 4:**
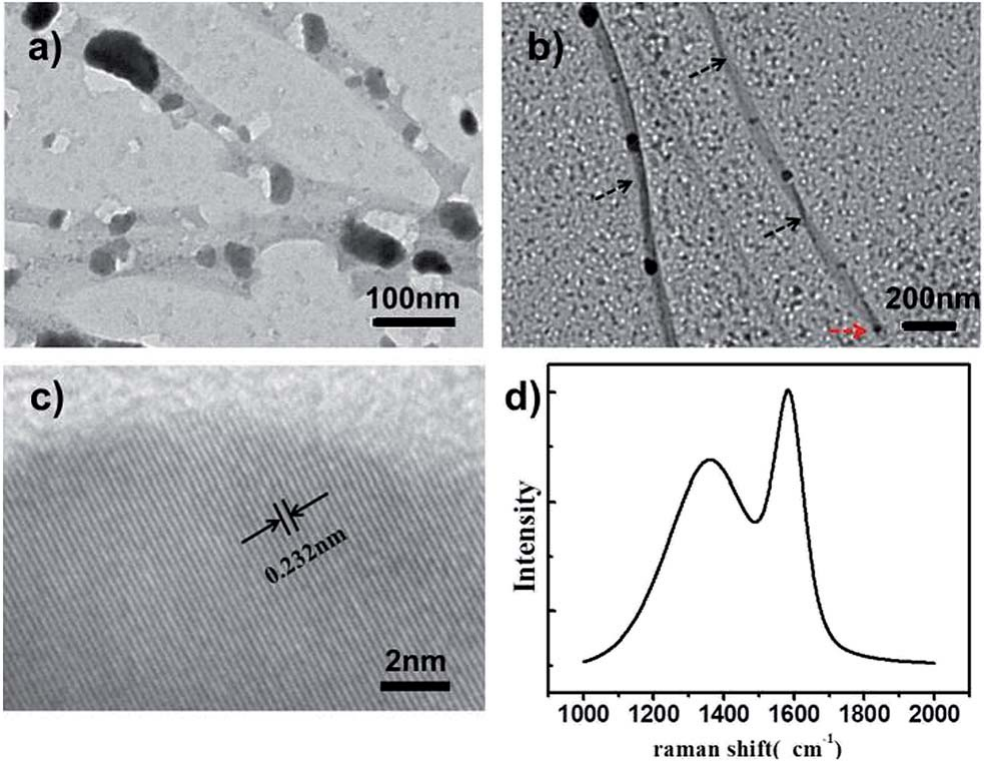
TEM images of the carbon nanostructures produced from **l-HDGA@HKUST-1** with thin (a) and thick (b) MOF shells; (c) HR-TEM image of the Cu nanoparticles decorated on the carbon surface; (d) Raman spectrum of the resultant carbon materials.

Helically nanostructured carbons have attracted great attention owing to their unique elasticity, chirality and electromagnetic wave absorption properties. However, the efficient production of enantiomerically pure carbon samples remains a big challenge.^15^ Very recently, MOFs have been demonstrated as novel precursors for the formation of carbon nanomaterials.^16^ Inspired by these reports, we attempted to utilize our prepared NMOFs with helical structures as precursors to fabricate helical nanocarbons. As a demonstration, **l-HDGA@HKUST-1** samples with different MOF shell thicknesses were carbonized at 600 °C under an inert N_2_ atmosphere for 30 min. Fig. 4 shows TEM images of the resultant products. For samples with a thin **HKUST-1** shell, only poorly defined perforated carbon nanostructures were obtained, as the thin shell of the **l-HDGA/HKUST-1** NMOF was not robust enough to maintain its morphology during carbonization (Fig. 4a). However, when **l-HDGA/HKUST-1** samples with thick MOF shells were used for carbonization, well-defined nanotubes with exclusively right-handed morphologies were obtained (Fig. 4b). Additionally, during the carbonization process at high temperature, the Cu^2+^ ions in **HKUST-1** could be reduced to copper nanoparticles with sizes of around 20 nm (Fig. 4c).^16*b*^ Fig. 4c clearly shows the typical lattice fringes of the copper metal. As clear evidence for the formation of the carbon nanostructures, Raman spectra for all products exhibited D and G bands at 1345 cm^–1^ and 1588 cm^–1^ (Fig. 4d), which are associated with the presence of disordered and graphitic carbon structures, respectively.^17^

The preliminary results described above are encouraging. Although this new transcription scheme was tested with **HKUST-1** and **MIL-100**, it can, in principle, be used as a general and effective transcription scheme for creating various nanostructured MOFs with well-defined morphologies. In this work, probably owing to the use of helical nanotubes as templates, the resultant NMOF helices are not remarkable when compared to those of the supramolecular templates. However, we believe that MOF-based nanomaterials with more exquisite helical morphologies could be substantially achieved when helical fibers are utilized for transcription. This part of the work is still ongoing in our lab. Over the past few decades, research into the fabrication of organized supramolecular assemblies in a controlled fashion has matured. A rich variety of discrete assemblies with well-defined sizes and novel morphologies have been achieved by modulation of the molecular structure of the amphiphiles and the assembly conditions.^6^ In addition to carboxyl groups, N-containing or amino acid units, which are very beneficial for anchoring MOF materials,^18^ could also be used as head groups for the development of amphiphilic molecules. These results and the knowledge gained could enable the construction of novel NMOF superstructures which are inaccessible by conventional methods.

## Conclusion

In summary, we report the first example of nanoscale metal–organic frameworks with helical morphologies, which may have interesting future optical and chemical sensing applications. Perhaps more importantly, this work represents a step towards the goal of harnessing the power of organized supramolecular aggregates to construct MOF superstructures with well-defined nanoscale features and exquisite morphologies, beyond what has been accomplished using conventional methods. Our results suggest that by using appropriate supramolecular nano-objects, one can achieve efficient control over the morphologies of the templated MOF materials. In view of the virtually unlimited tunability of supramolecular assemblies, we believe that more complicated MOF nano- or superstructures should be accessible with this approach, which could significantly extend the applications of MOFs by exploiting their size and morphology related properties.
