# Supplementary material for: Helically structured metal–organic frameworks fabricated by using supramolecular assemblies as templates
Source: Chem Sci. 2014 Dec 23;6(3):1910–6. doi: 10.1039/c4sc03278k (PMC5506886; doi:10.1039/c4sc03278k)
Supplement: Supplementary file 1 [file SC-006-C4SC03278K-s001.pdf]

Electronic Supplementary Material (ESI) for Chemical Science.
This journal is © The Royal Society of Chemistry 2014

Supporting Information

Helically Structured Metal-Organic Frameworks Fabricated by Using Supramolecular Assemblies as Templates

Hui Wang,†[a] Wei Zhu,†[a] Jian Li,[a] Tian Tian,[a] Yue Lan,[a] Ning Gao,[a] Chen Wang,[a] Meng Zhang,[a] Charl F. J. Faul[b] and Guangtao Li*[a]

[a] Department of Chemistry and Key Lab of Organic Optoelectronics & Molecular Engineering, Tsinghua University, 100084 Beijing, China.

[b] School of Chemistry, University of Bristol, Cantock's Close, Bristol BS8 1TS, U. K.

1. Experimental Section

1.1 *Chemicals*

The amphiphile D-HDGA and L-HDGA was synthesized according to a previous published procedure.[S1] All solvents and chemicals were purchased from Alfa and used as received. All solvents and chemicals were of reagent quality and were used without further purification unless specifically mentioned.

1.2 *Instrumentation*

^{1}H NMR spectra were obtained using a JEOL JNM–ECA300 at 300 MHz. Electrospray ionization mass spectrometry (ESI-MS) was obtained by means of Bruker ESQUIRE-LC spectrometer. X-ray diffraction (XRD) spectra were recorded on a D/max- RB (Japan, Rigaku) diffractometer with monchromatized Cu Kα radiation (λ=0.15418 nm), operating at 40 Kv and 120 mA. Data were obtained with a scanning rate of 4.0° min^{-1}. The size and morphology of as-synthesized samples were determined by using a Hitachi model H-7650 transmission electron microscope and a JEM-2010 high-resolution transmission electron microscope. CD spectra were obtained using JASCO J-810 spectrophotometers. The Raman spectrum was recorded on a Renishaw Raman microscope with a 514 nm wavelength laser.

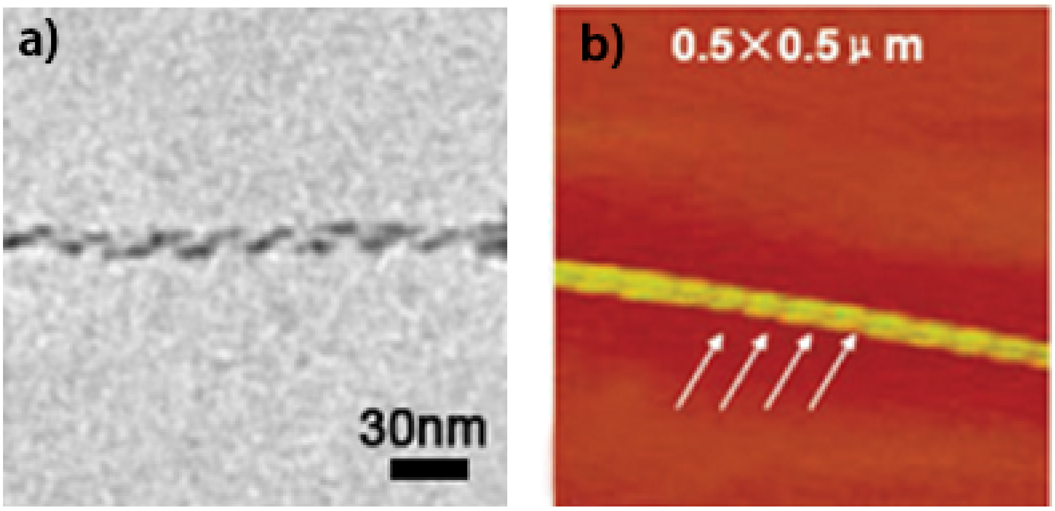

Figure S1: HR-TEM image (a) of the supramolecular aggregate formed from self-assembly of L-HDGA; and the AFM image (b) cited from the reported work (M. H. Liu et al. *Chem.Comm.,* 2010, **46**, 7178).

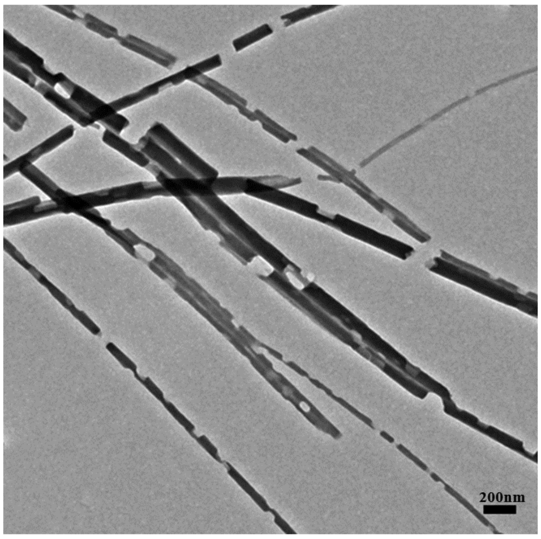

Figure S2: TEM image of as-prepared HKUST-1 products under concentrated gelation conditions.

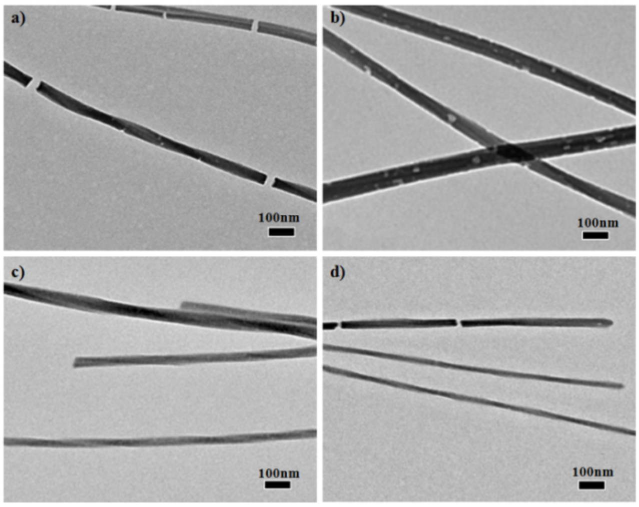

Figure S3: TEM images of the as-prepared helical HKUST-1 templated by a series of diluted hydrogels, which were prepared by diluting the original hydrogels with different ethanol-water mixtures. [Volume percentage of ethanol: a) 37.5%, b) 25%, c) 12.5%, d) 0.]

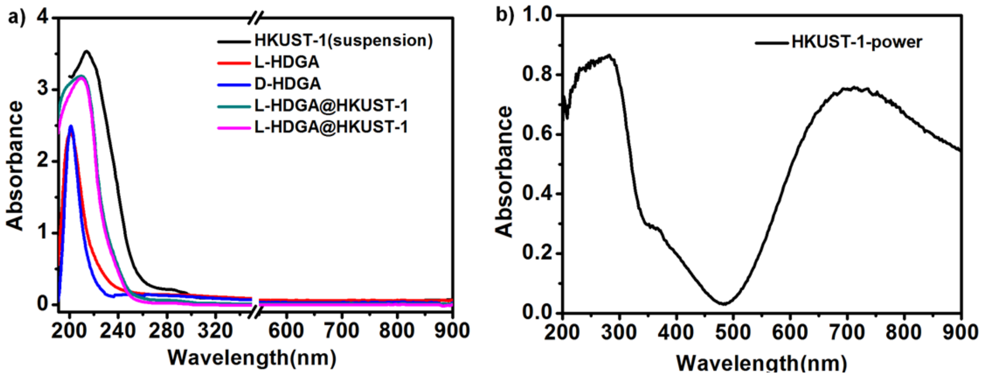

Figure S4: UV-Vis spectra of the L/D-HDGA templates, the as-prepared helical L/D-HDGA@HKUST-1(a) and the HKUST-1 power (b).

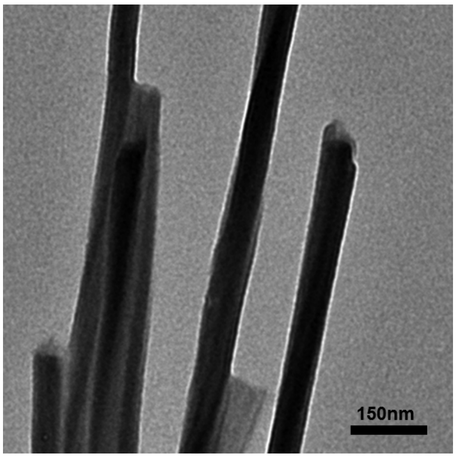

Figure S5: TEM images (b-d) of the resultant helical 4-L-HDGA@HKUST-1.

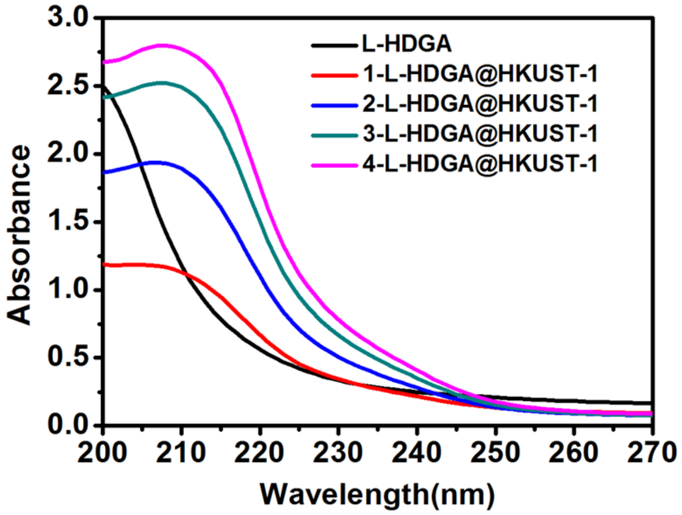

Figure S6: UV-Vis spectra of the L-HDGA-based template and the corresponding right-handed helical HKUST-1 with increased addition of MOF precursor.

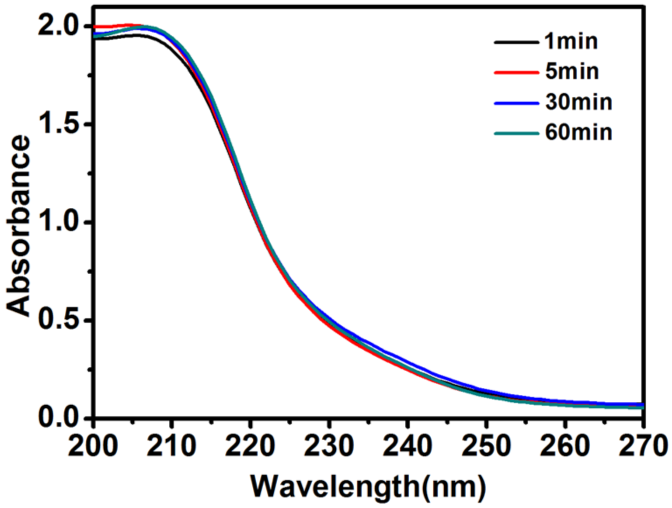

Figure S7: UV-Vis spectra of the resultant helical 2-L-HDGA@HKUST-1 with increased reaction time.

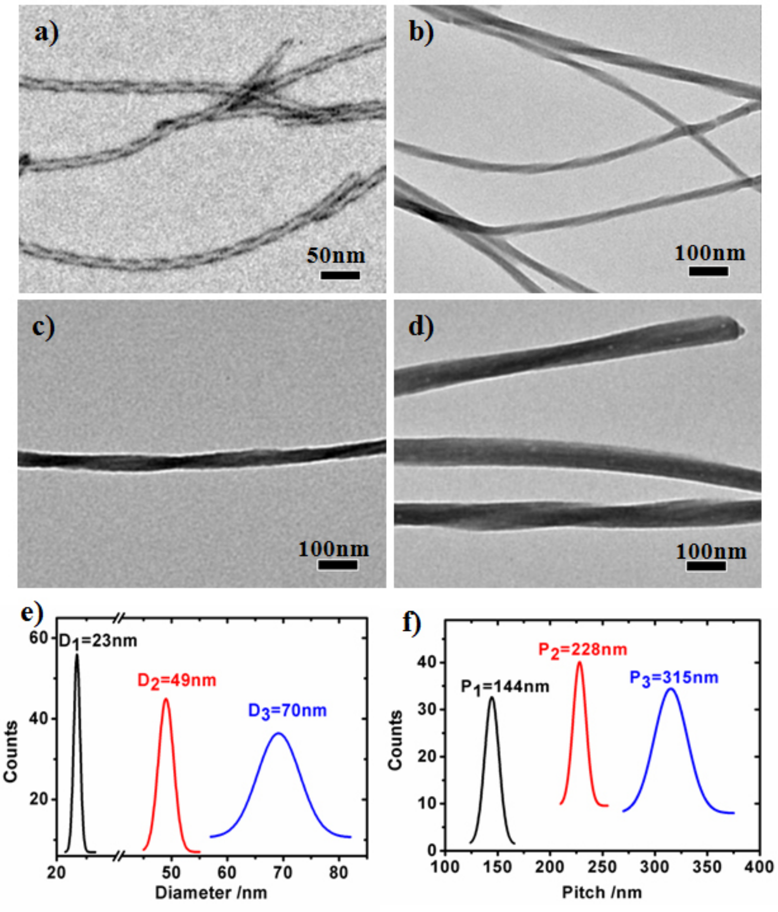

Figure S8: TEM images of left-handed helices prepared from self-assembly of D-HDGA (a); TEM images of the resultant helical D-HDGA@HKUST-1 with increased addition of MOF precursor (b-d); the evolution of diameter (c) and pitch (d) of the resultant helical D-HDGA@HKUST-1 with increased addition of MOF precursor.

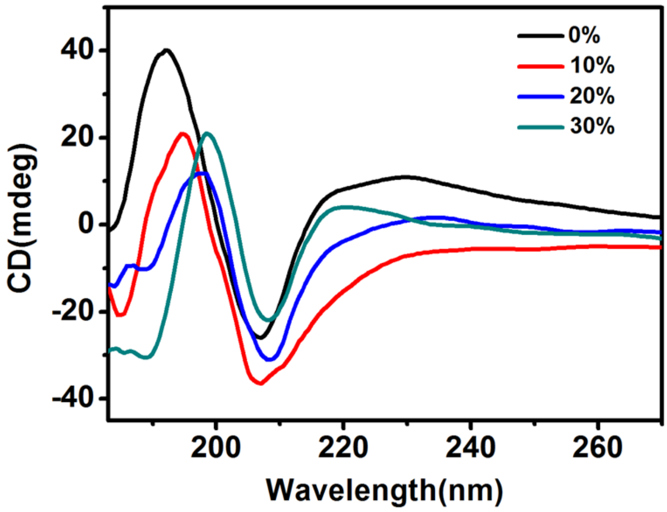

Figure S9: Circular dichroism spectra of the L-HDGA solution diluted with 4 mL aqueous solution containing different amounts of ethanol (volume percentage of ethanol: 0, 10%, 20%, and 30%).

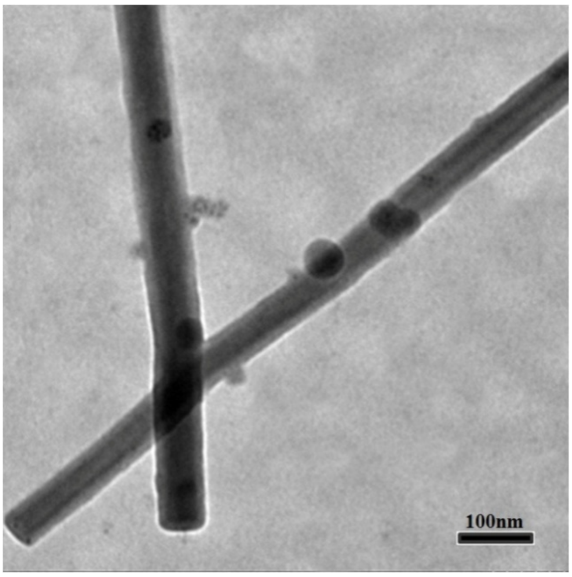

Figure S10: TEM image of HKUST-1 nanotubes prepared by wipping off L-HDGA template.

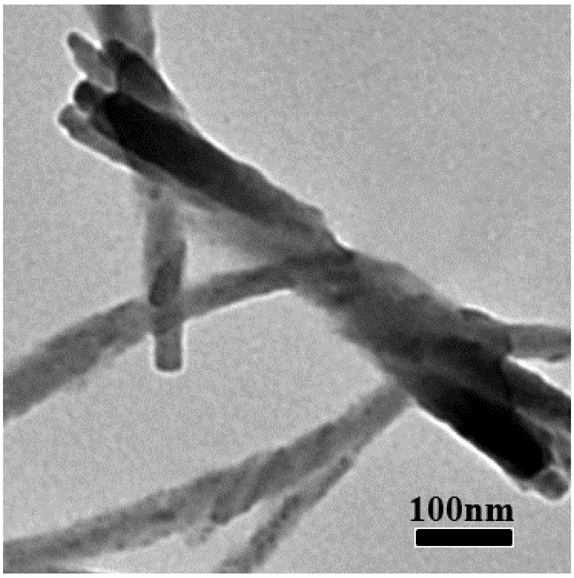

Figure S11: TEM image of single helical MIL-100 nanostructures.

References

S1. J. Jiang, T. Y. Wang, and M. H. Liu, *Chem.Comm.,* 2010, **46**, 7178.
